# Deciphering the functional role of EGR1 in Prostaglandin F2 alpha induced luteal regression applying CRISPR in corpus luteum of buffalo

**DOI:** 10.1186/s40659-021-00333-7

**Published:** 2021-03-12

**Authors:** Meeti Punetha, Sai Kumar, Avishek Paul, Bosco Jose, Jaya Bharati, Arvind Sonwane, Jonathan A. Green, Kristin Whitworth, Mihir Sarkar

**Affiliations:** 1 Physiology & Climatology Division, ICAR-Indian Veterinary Research Institute, Izatnagar, Bareilly, Uttar Pradesh 243122 India; 2 Animal Physiology, ICAR-National Research Centre on Pig, Guwahati, Assam India; 3 Division of Animal Genetics, ICAR-Indian Veterinary Research Institute, Izatnagar, Bareilly, Uttar Pradesh 243122 India; 4 Division of Animal Science, University of Missouri-Columbia, Columbia, MO USA

**Keywords:** Buffalo, Corpus luteum, EGR, CRISPR/Cas9, Luteolysis

## Abstract

**Background:**

PGF2α is essential for the induction of the corpus luteum regression which in turn reduces progesterone production. Early growth response (EGR) proteins are Cys2-His2-type zinc-finger transcription factor that are strongly linked to cellular proliferation, survival and apoptosis. Rapid elevation of EGR1 was observed after luteolytic dose of PGF2α. EGR1 is involved in the transactivation of many genes, including TGFβ1, which plays an important role during luteal regression.

**Methods:**

The current study was conducted in buffalo luteal cells with the aim to better understand the role of EGR1 in transactivation of TGFβ1 during PGF2α induced luteal regression. Luteal cells from mid stage corpus luteum of buffalo were cultured and treated with different doses of PGF2α for different time durations. Relative expression of mRNAs encoding for enzymes within the progesterone biosynthetic pathway (*3βHSD*, *CYP11A1* and *StAR*);* Caspase 3*; *AKT* were analyzed to confirm the occurrence of luteolytic event. To determine if EGR1 is involved in the PGF2α induced luteal regression via induction of TGFβ1 expression, we knocked out the EGR1 gene by using CRISPR/Cas9.

**Result:**

The present experiment determined whether EGR1 protein expression in luteal cells was responsive to PGF2α treatment. Quantification of *EGR1* and *TGFβ1* mRNA showed significant up regulation in luteal cells of buffalo at 12 h post PGF2α induction. In order to validate the role of PGF2α on stimulating the expression of *TGFβ1* by an EGR1 dependent mechanism we knocked out EGR1. The EGR1 ablated luteal cells were stimulated with PGF2α and it was observed that EGR1 KO did not modulate the PGF2α induced expression of *TGFβ1*. In PGF2α treated EGR1 KO luteal cell, the mRNA expression of *Caspase 3* was significantly increased compared to PGF2α treated wild type luteal cells maintained for 12 h. We also studied the influence of EGR1 on steroidogenesis. The EGR1 KO luteal cells with PGF2α treatment showed no substantial difference either in the progesterone concentration or in *StAR* mRNA expression with PGF2α-treated wild type luteal cells.

**Conclusion:**

These results suggest that EGR1 signaling is not the only factor which plays a role in the regulation of PGF2α induced TGFβ1 signaling for luteolysis.

## Background

The corpus luteum is a transient endocrine organ which regulates ovarian cyclicity and maintains pregnancy by acting as the primary source of progesterone production (P4) in bovine and numerous domestic species. The development of the corpus luteum and its endocrine function are dependent on angiogenesis and vasculogenesis. The mature corpus luteum is comprised of two steroidogenic cell populations, the large and small luteal cells, which are derived from ruptured follicular granulosa and thecal cell, respectively [1]. These large and small luteal cells have ProstaglandinF2α (PGF2α) receptors, which upon binding by PGF2α, can induce luteal regression [2]. Corpus luteum regression leads to a decline in circulating progesterone concentration, which promotes follicular growth and a resumption of reproductive cyclicity [3].

In bovine, PGF2α stimulation of the ovary suppresses luteotropic factors and stimulates luteolytic factors [4]. Indeed, PGF2α is the primary hormone initiating CL regression in cattle [3]. Previous studies in cattle, pig, human and rat has indicated that PGF2_α_ activates ERK (extracellular-signal-regulated kinase) signaling in luteal cells [5, 6]. PGF2α binds to a G-protein coupled receptor on the target cell and activates PKC signaling pathway [7]. PKC further activates the MEK1/Raf/ERK1/2 signaling pathway in luteal cells [5, 8]. Upon activation, ERK translocates to the nucleus where the binding of SRF/Elk-1 is promoted which subsequently regulates EGR1 [9].

Early growth response (EGR) proteins are Cys2-His2-type zinc-finger transcription factor family members. They are represented by the following genes: EGR1, EGR2, EGR3 and EGR4. EGR1, 2 and 3 are transcriptional activators whereas EGR4 is regarded as a transcriptional repressor [10]. EGR proteins specially bind to the major groove of DNA at GC-rich DNA recognition sites to alter the transcription of various genes necessary for differentiation and mitogenesis in the various tissues [11]. The best studied member of this family is EGR1. EGR1 is an 80-kDa DNA binding protein which functions as a convergence point for many signaling cascades. Extracellular signaling from growth factors, hormones and cytokines induces EGR1 expression in a rapid and transitory manner [12]. Therefore, EGR1 gene is used for studying stimulus-transcription pairing [13].

EGR1 binds to regulatory regions of target genes. Indeed, EGR1 plays an essential role in the transcriptional regulation of genes whose protein products regulate CL regression [14]. One well described example is its ability to enhance transcription of transforming growth factor β1 (TGFβ1) [15, 16]. When bovine luteal cells are treated with luteolytic doses of PGF2_α_, the RAF/ERK/MAPK kinase pathway is recognized as a primary signal, which induces EGR1 that leads to increase of TGFβ1 expression [17]. Expression of TGFβ1 is associated with CL regression through inhibition of luteal angiogenesis [18], increasing apoptosis, tissue remodeling [42] and suppression of progesterone production [17, 19]. The expression of TGFβ1 in CL is up regulated when administered with PGF2α during luteal regression [5, 17, 20–22].

Previous studies, indicates that EGR1 plays an important role in PGF2α induced expression of TGFβ1 which in turn plays an important role during luteal regression. However, studies on functional role of EGR1 on PGF2α induced luteolysis in buffalo is not available. Hence, for the purpose, we knocked out (KO) the *EGR1* gene via CRISPR/Cas 9 genome editing technology to examine how loss of EGR1 would influence *TGFβ1* expression after PGF2α treatment in luteal cells.

## Materials and methods

All procedures and experimental protocols followed relevant safety guidelines and regulations.

### Collection of CL

Sixty ovaries from healthy buffalo cows with normal reproductive tracts to extract six CL per group for studies have been obtained from a local abattoir in 1× phosphate-buffered saline maintained at 37 °C. Only mid-luteal stage corpus luteum was used in the present experiment. The selection of mid stage CL was conducted out as per our laboratory’s pre-established protocol [23].

### Culture of luteal cells

**Fig. 1:**
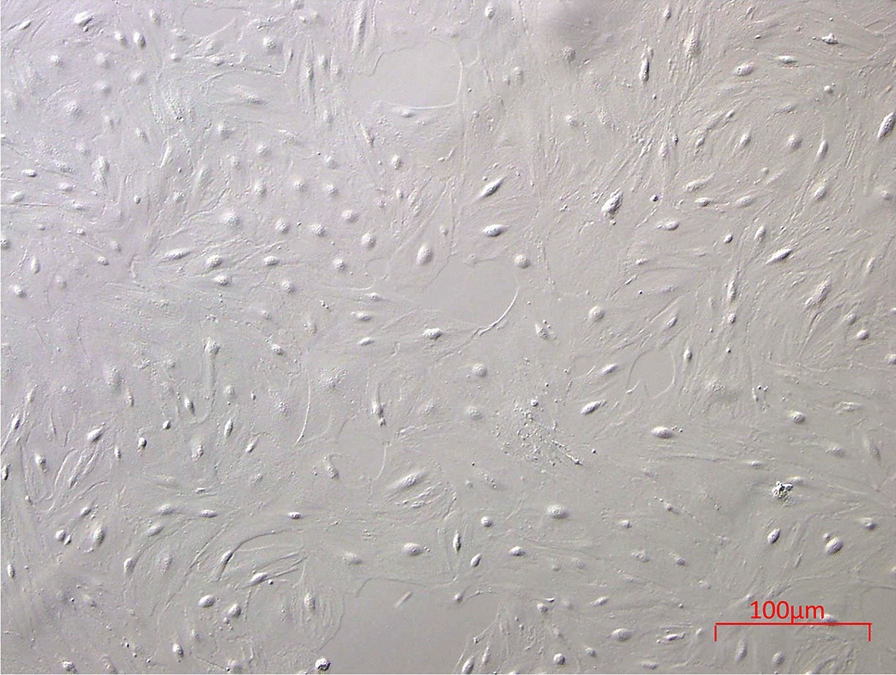
Culture of attached luteal cells (×10 magnification)

The luteal cells were cultured by using a pre-established protocol from our laboratory [24]. In brief, mid stage CLs were excised from the ovary and were chopped using BP blades (Bard-Parker Surgical Blade). The minced luteal tissue was washed thrice at 150×*g* for 5 min at room temperature with washing medium (Dulbecco’s modified Eagle’s medium DMEM/F12 medium (SH30023.01; Hyclone, Thermo Scientific) and 1% antibiotic–antimycotic solution (Gibco; Thermo Scientific). After washing, the minced cells containing luteal, endothelial, pericytes and fibroblasts cells were digested in DMEM/F12 medium containing collagenase 2 mg/ml (MP Biomedicals), DNaseI 25 mg/ml (MP Biomedicals) and 0.5% bovine serum albumin (BSA). Washed cells were incubated in digestion media for 45 min twice in an orbital shaker incubator maintained at 37 °C. The digested cells were then filtered through a 70 µm filter (Molsheim, France) and were washed in culture medium containing DMEM/F12 medium, 10% fetal bovine serum (FBS; Sigma-Aldrich) and 1% antibiotic–antimycotic solution. Cell viability was assessed by using Trypan blue vital staining (Sigma-Aldrich). Cells were then plated out at 1.5 × 10^5^ viable cells per well in a 24-well plate with 1 mL culture medium and were maintained at 37 °C in a humidified CO2 (5%) incubator. Once plated, cells attached and grown until 75–80% confluency (Fig. 1). At this point, the medium was replaced with the fresh medium containing PGF2α analog, (0.1, 1 and 10 µg/mL) [25]; cultures were maintained for 4, 8, 12 h in replicates of six each group. Control cells were grown in medium without hormone or growth factor. After the specified period of time, spent medium were collected and kept at − 20 °C until used for progesterone estimation by radioimmunoassay (RIA). Cells were trypsinized and total RNA was extracted via Qiazol (Qiagen).

### Production of EGR1 knock out (KO) luteal cells

Production of EGR1 KO luteal cells was carried out by CRISPR/Cas9 by the method described earlier [24, 26]. The CRISPR/Cas9 components (single guide RNA and Cas9) were delivered via lipofection into the luteal cells GeneArt Genomic Cleavage Detection Kit (Invitrogen) was used to validate the EGR1 KO and the cleavage efficiency was calculated by the following formula [27]: Cleavage efficiency = [(sum of cleaved band intensities)/(sum of cleaved and parental band intensities)] × 100%

### Treatment of EGR1 KO luteal cells with PGF2α

Following the above described procedure, all the EGR1 KO cells were cultured and grown until 75–80% confluency was reached. Thereafter, the medium was replaced with fresh medium with or without a PGF2α analog (10 µg/mL). Cells were cultured for an additional 12 h. Afterwards, the spent medium was collected and used for progesterone estimation. The cells were trypsined and total RNA was isolated.

### Primers

The primers *EGR1*, *AKT*, *Caspase3*, *StAR*, *TGFβ1*, *3βHSD*, *CYP11A1* were designed using Gene Tool (online trial version), DNAStar (online trial version), and Oligo Analyser (open access tool) software. For the study some published were used which includes 40S ribosomal protein S15 (*RPS15A*) [24] and von willebrand factor (*vWF*). In Table 1 is given the list of primers used for the analysis.

**Table 1:** Target gene, primer sequences and amplicon length used in the qPCR and Knockout study

Gene	Sequences of nucleotide (5′–3′)	Amplicon length (bp)	EMBL accession no. or reference
EGR 1	Forward: AGCTGTGCAGTGCAGTCCAACGAC Reverse: TAGTCGGGGATCATGGGAACCTG	194	XM_006070841.2
vWF	Forward: ATCGTAGGGGACTTCCAAGGTGG Reverse: CGGTCTCCAGGTATAGCCCTCTGG	154	52
AKT	Forward: AAACCGTTACCTTGCTATG Reverse: TGCCCAGTTCGTTTCAGT	159	NM_174056. 3
RPS15A	Forward: AGGGCTGGGAAAATTGTTGTGAA Reverse: TGAGGGGATGGGAGCAGGTTAT	104	24
CYP11A1	Forward: AGACTTGGAGGGACCATGTAGC Reverse: TGCCTGGGTAATTCCTAAATTC	117	XM_025271874.1
TGF β1	Forward: AACAATTCCTGGCGCTACCTC Reverse: TGCCGCACAACTCCAGTG	92	XM_006052063.2
3βHSD	Forward: AATCCGGGTGCTAGACAAAGT Reverse: CACTGCTCATCCAGAATGTCTC	111	XM_006049357.2
StAR	Forward: CTGCGTGGATTAACCAGGTTCG Reverse: CCAGCTCTTGGTCGCTGTAGAG	84	XM_006054485.2
EGR1 SgRNA	Forward:TAATACGACTCACTATAGGTCCATGGTGGGCGAATG Reverse: TTCTAGCTCTAAAACCATTCGCCCACCATGGAC	–	26
EGR1 genomic detection	EGR1 Forward: CTACCCCAGCCTCGGTAGCA EGR1 Reverse: TCAGGTGCTCGTAGGGCTGC		

### Quantitative real time PCR analysis

QIAzol reagent (QIAGEN) was used to extract total RNA from the cultured luteal cells. The purity of RNA was determined in Nanodrop spectrophotometer A260/A280. The integrity of the total RNA was confirmed with electrophoresis of the agarose gel. Total RNA (1 μg) was reversed transcribed to cDNA using a cDNA synthesis kit (ThermoFisher Scientific) as specified by the manufacturer using oligo dT primers at 42 °C for 60 min. The qPCR was performed using the Maxima SYBR Green qPCR kit (Thermo Scientific). Each sample was run in triplicate in a 25 μL reaction mixture consisting of 12.5 μL SYBR green mix, 0.5 µL each of 0.3 µM forward and 0.3 µM reverse primers, 1 µL cDNA and 10.5 µL nuclease-free water. The following general qPCR protocol was followed: initial denaturation for 10 min at 95 °C followed by 40 denaturation cycles at 95 °C for 15 s, annealing and extension at 60 °C for 60 s. Real-time PCR’s efficiency was calculated by amplification of a standardized series of dilution, and slopes were achieved.

### Hormone determination

The concentration of Progesterone (P4) in spent media of cultured luteal cell was assessed by P4^125^I RIA kit (Immunotech) according to the package recommendations. The coefficients of variation for intra and inter assay were 6.5% and 7.2% respectively.

### Statistical analysis

All experimental data are shown as means. The software SPSS.22 was used to determine the statistical significance of differences in transcriptional regulation of all genes and P4 concentrations (treatment dose and time interval) by two way analysis of variance followed by Tukey’s honestly significant difference (HSD) test as a multiple comparison test. Differences were considered to be significant at p < 0.05.

## Results

### Effects of Prostaglandin F2α on luteal cell steroidogenesis in buffalo

**Fig. 2:**
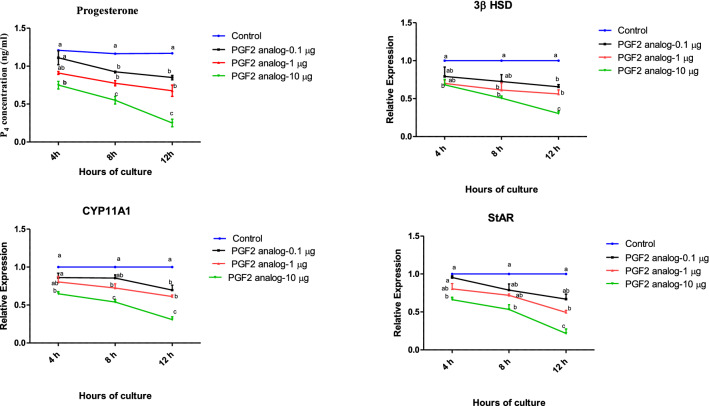
Effect of PGF2α (0.1, 1 and 10 µg/mL) over a period of 4, 8, 12 h (n = 6/condition) on the concentration of progesterone (**a**) and mRNA expression of *3βHSD* (**b**), *CYP11A1* (**c**) and *StAR* (**d**), in in vitro luteal cell culture. *3βHSD* 3beta hydroxyl steroid dehydrogenase, *CYP11A1* cytochrome P450 side chain cleavage subfamily A1, *StAR* steroidogenic acute regulatory protein; (*p* < 0.05), statistically significant

To understand the effect of PGF2α in corpus luteum, luteal cells derived from the mid stage CL of buffalo were treated with PGF2α. To determine the effect of PGF2α on steroidogenesis, the progesterone concentration in the spent media and the relative expression of mRNAs encoding for enzymes within the progesterone biosynthetic pathway (*3βHSD*, *CYP11A1* and *StAR*) were analyzed. The study revealed a significant decrease in the progesterone concentration after 12 h of culture in a dose-dependent manner (p < 0.05; Fig. 2a). This finding was further supported by the study of mRNA expression of *3βHSD*, *CYP11A1* and *StAR*. Of the three concentrations tested, 10 μg/mL PGF2α showed significant down regulation of progesterone biosynthetic pathway enzymes at 4, 8, 12 h of culture in comparison with the other doses (p < 0.05; Fig. 2b–d).

### Effects of Prostaglandin F2 α on luteal cell viability and angiogenesis in buffalo

**Fig. 3:**
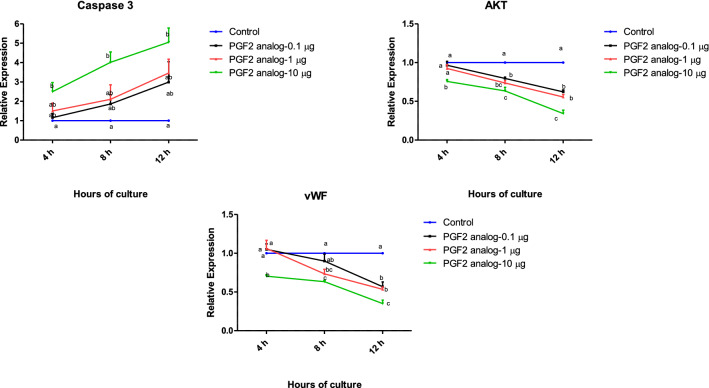
Effect of PGF2α (0.1, 1 and 10 µg/mL) over a period of 4, 8, 12 h (n = 6/condition) on mRNA expression of *Caspase 3* (**a**), *AKT* (**b**) and *vWF* (**c**) in in vitro luteal cell culture. vWF, von Willebrand factor; (*p* < 0.05), statistically significant

In order to understand the effect of PGF2α on luteal cell viability, mRNA expression of *Caspase 3* and *AKT* was studied. The mRNA expression study of apoptotic gene *Caspase 3* was significantly up regulated in a time dependent manner at the highest dose of PGF2α (p < 0.05; Fig. 3a) as compared to other doses. However, the mRNA abundance of the cell proliferation gene, *AKT*, showed significant down regulation in all three doses of PGF2α that were tested at 8 and 12 h (p < 0.05; Fig. 3b). Similarly, the expression of the angiogenic marker, von Willebrand factor (*vWF*), showed significant down regulation at 12 h of culture in all three doses of PGF2α that were tested (p < 0.05; Fig. 3c).

### PGF2α induces the expression of EGR1 and TGFβ1

**Fig. 4:**
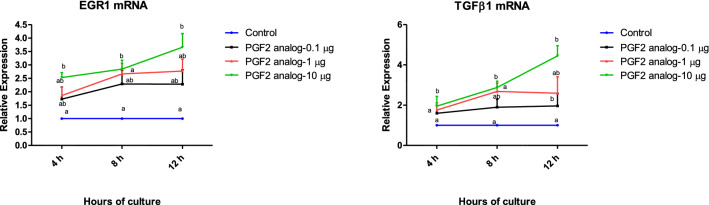
Effect of PGF2α (0.1, 1 and 10 µg/mL) over a period of 4, 8, 12 h (n = 6/condition) on mRNA expression of *EGR1* (**a**) and *TGFβ1* (**b**) in in vitro luteal cell culture. EGR1, Early Growth response factor 1; *TGF β1* transforming growth factor beta 1; (*p* < 0.05), statistically significant

The present experiment determined whether EGR1 protein expression in luteal cells was responsive to PGF2α treatment. The mRNA encoding for EGR1 significantly increased at the highest dose of PGF2α (10 µg/mL) in a time dependent manner (p < 0.05; Fig. 4a) as compared with the other doses. Similarly, the effect of PGF2α at 10 μg/mL concentration also showed a significant increase in TGFβ1 mRNA at 4, 8, 12 h of culture as compared to other doses which did not show significant up regulation at 4 and 8 h of culture (p < 0.05; Fig. 4b).

### Determining the effect of knocking out EGR1 on PGF2α induced expression of TGFβ1

**Fig. 5:**
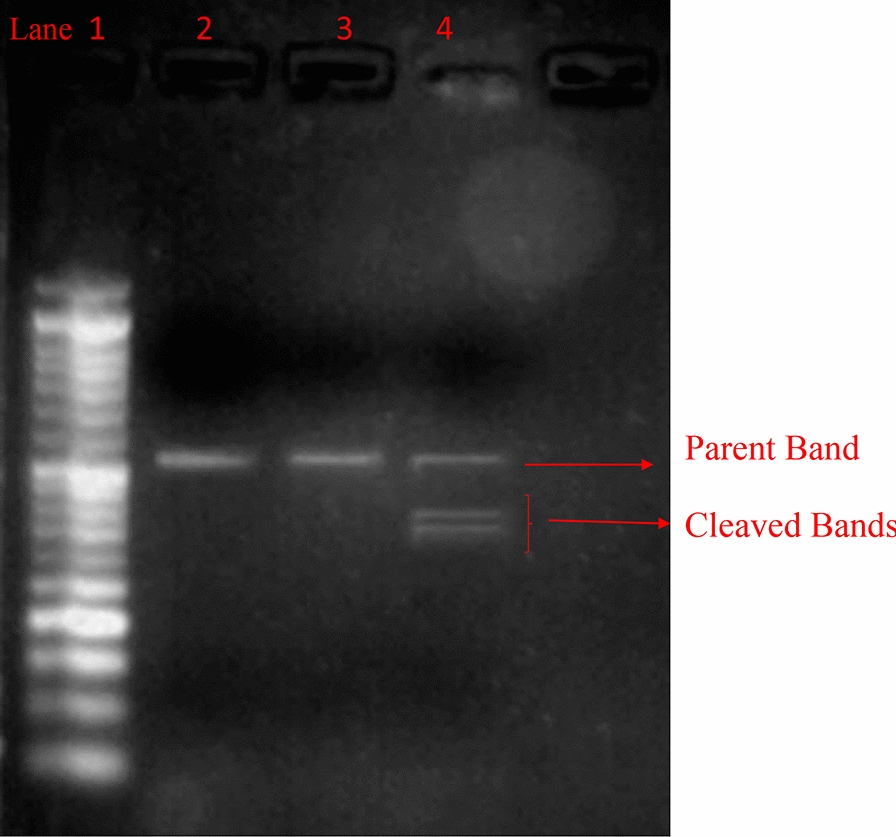
Gel image of genomic cleavage detection assay of luteal cells transfected with Cas9 and EGR1 SgRNA using Lipofectamine 2000. Lane 1, 50 bp DNA ladder; Lane 2, negative control sample for EGR1 gene; Lane 3 sample without T7/E1 enzyme, Lane 4: sample, showing parent and both the cleaved bands after addition of T7/E1 enzyme

**Fig. 6:**
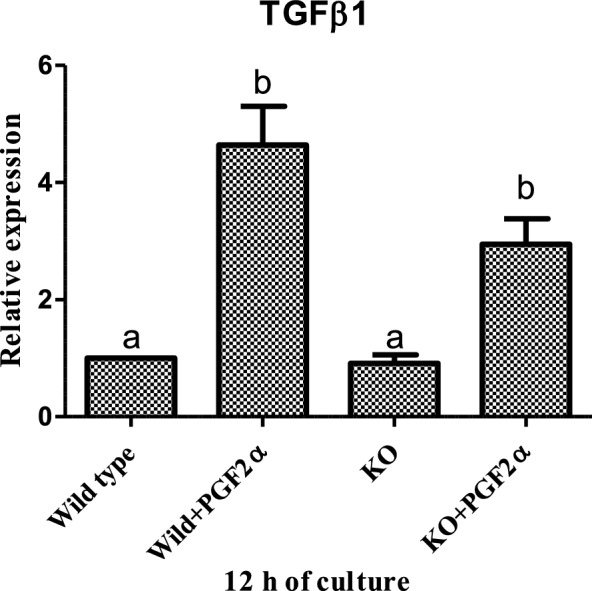
Effect of PGF2α (10 µg/mL) on mRNA expression of *TGF β1* in MLC and EGR1 KO MLC at 12 h (n = 6/condition). All values are shown as mean ± SEM. Different superscripts denote statistically different values (P < 0.05). EGR 1 KO, Early Growth Response factor 1 Knock out; MLC, Mid stage luteal cells; (*p* < 0.05), statistically significant

In order to validate the role of PGF2α on stimulating the expression of *TGFβ1* by an EGR1 dependent mechanism we knocked out EGR1 via CRISPR/Cas9 genome editing technology. The confirmation of EGR1 knock out was validated by T7E1 Genomic Cleavage Detection Kit, in which the efficiency of knock out of EGR1 was found to be 70% (Fig. 5). In the present study, we observed that PGF2α induced *TGFβ1* expression in EGR1 KO luteal cells. The ablation of EGR1 did not modulate the PGF2α induced expression of TGFβ1 (Fig. 6).

### Determining the effect of knocking out EGR1 on PGF2α induced luteal cell viability and function

**Fig. 7:**
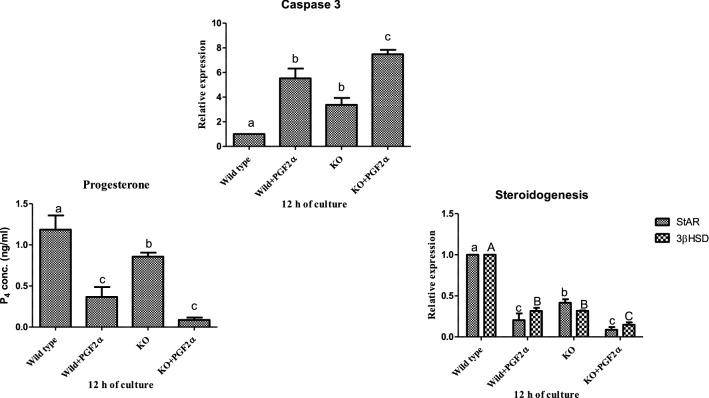
Effect of PGF2α (10 µg/mL) on transcriptional abundance of *Caspase 3* (**a**), Progesterone concentration (**b**) and on transcriptional abundance of *StAR*, *3βHSD* (**c**) in MLC and EGR1 KO MLC at 12 h (n = 6/condition). All values are shown as mean ± SEM. Different superscripts denote statistically different values (P < 0.05). *EGR1 KO* Early Growth Response factor 1 Knock out, *MLC* mid stage luteal cells, *StAR* steroidogenic acute regulatory protein, *3βHSD* 3beta hydroxyl steroid dehydrogenase; (*p* < 0.05), statistically significant

The study revealed significant difference in the mRNA expression of *Caspase 3* in EGR1 KO luteal cell and wild type luteal cells maintained for 12 h (p < 0.05; Fig. 7a) in in vitro cell culture. In PGF2α treated EGR1 KO luteal cells, the mRNA expression of *Caspase 3* was significantly increased compared to PGF2α treated wild type luteal cells maintained for 12 h (p < 0.05; Fig. 7a). We also studied the influence of EGR1 on steroidogenesis and its pathway. The progesterone concentration of EGR1 KO luteal cells in the spent media showed significant differences with the wild type luteal cells (p < 0.05; Fig. 7b). The steroidogenic enzymes also showed significant difference between the wild type luteal cells and EGR1 KO luteal cells (p < 0.05; Fig. 7c). However, the EGR1 KO luteal cells with PGF2α treatment showed no substantial difference either in the progesterone concentration or in *StAR* mRNA expression with PGF2α-treated wild type luteal cells (Fig. 7b, c).

## Discussion

Dramatic morphological and functional changes occur during the life span of CL. In the absence of pregnancy, the mature CL eventually undergoes functional and structural regression, which is necessary for the initiation of a subsequent ovarian cycle. In cattle and related species, PGF2α is the primary hormone initiating CL regression [3]. In fact, exogenous administration of PGF2α can initiate luteolysis [28]. In buffalo, the functional phase of luteolysis lasts for approximately 12 h and is followed by structural changes in the luteal tissue; both of these changes are reflected by declining progesterone concentration in the blood [29]. In the present study, the progesterone concentration in the spent media was significantly down regulated upon treatment with PGF2α (10 µg/mL) for 4, 8 and 12 h duration (Fig. 2a). These findings are consistent with prior studies wherein progesterone concentration decreased during the first 12 h after PGF2α administration [4]. The decreased P4 production was also consistent with the observed mRNAs expression for *3βHSD*, *CYP11A1* and *StAR* (Fig. 2b–d). Once again, the results were in accordance with previous studies in which PGF2α decreased progesterone concentration along with the down regulation of steroidogenic enzymes [2, 14]. PGF2α administration produced a 30% decrease in plasma P4 concentration at 30 min and 2 h which further declined at 12 h [30]. Thus, the decrease in progesterone concentration in the spent media in our experiment confirmed the occurrence of luteolysis.

The structural involution of CL during its regression involves apoptosis, or cell programmed death [6, 31] which can be evaluated by the ratio of pro-apoptotic to anti apoptotic proteins [32]. The transcriptional abundance of *Caspase 3* and *BAX* within CL are increased in buffalo CL treated with PGF2α, which is also correlated with declining progesterone [33]. In the present study, the expression of *Caspase 3* increased, along with a decline in *AKT* mRNA, in a dose and time dependent manner after PGF2α treatment (Fig. 3a, b). We have demonstrated earlier that the effect of PGF2α at 10 μg/mL significantly up regulated the expression of *Caspase 3* at 72 h of culture as compared to other doses at 24 and 48 h [25] and it has also been reported that *Caspase 3* is an essential mediator of apoptosis during luteolysis [34]. Thus, the increased expression of pro-apoptotic *Caspase 3* during CL regression might be the ultimate reason for the disruption of steroidogenic cells which eventually leads to cell death.

Angiogenesis and vascularization of CL are required to supply nutrients and hormones for maintaining CL growth and survival [35]. Prostaglandin F2α (PGF2α) can inhibit angiogenesis to promote luteolysis [35]. Luteolysis is marked by a reduction in blood vessel density along with the degeneration and disappearance of luteal cells [36]. In the present study the angiogenic marker, vWF was significantly down regulated by PGF2α treatment (Fig. 3c). The reduced angiogenesis could be due to reduced expression of the angiogenic growth factors, FGF2 and VEGF, and increased expression of anti-angiogenic factors, such as thrombospondin, which in turn destabilizes luteal vessel and reduces hormonal levels [37]. Most importantly, work earlier conducted in our laboratory shows that PGF2α contributed to the luteolytic cascade by promoting the pro-apoptotic and anti angiogenic activity of Thrombospondin1 [25]. Other published reports have shown that the capillary degeneration and endothelial cell death are associated with PGF2α induced luteolysis [38–41].

Transforming growth factor β1 (TGFβ1) in luteal cell cultures is known for its potential involvement in apoptosis, tissue remodeling [42] and decreasing progesterone secretion [19]. The expression of *TGFβ1* mRNA was found to be up regulated in CL when PGF2α was administered to induce luteal regression in several species viz bovines [17, 20–22], mouse [43] etc. TGFβ1 facilitates luteal regression by disrupting the angiogenic potential of bovine microvascular endothelial cells [18]. In the present study, we have shown that a luteolytic dose of PGF2α induced *TGFβ1* mRNA in the buffalo luteal cells in vitro. Thus, PGF2α plays a significant role in regulating the production of TGFβ1 during regression of the CL.

In the present investigation, EGR1 expression was found to be elevated in luteal cell when treated with PGF2α in a dose dependent manner. In an earlier study conducted in bovines administration of PGF2α increased *EGR1* mRNA and EGR1 protein in CL [17]. During luteal regression in many species studied so far EGR1 plays a crucial role in the transcriptional regulation of genes [14]. EGR1 is a transcription factor that binds to the regulatory regions of many genes known to be involved in the regressive changes in CL during PGF2 α induced luteolysis; TGFβ1 is one of the regulated targets [15, 16]. And this prompted us to validate the role of PGF2α on stimulating the expression of *TGFβ1* by an EGR1 dependent mechanism and for this purpose EGR1 was knocked out via CRISPR/Cas9 genome editing technology. In our study, we observed that PGF2α induces *TGFβ1* expression in KO luteal cells and the ablation of EGR1 did not modulate the PGF2α induced expression of *TGFβ1*. These finding suggests the presence of other signaling pathways that might be involved in PGF2α induced *TGFβ1* expression. PGF2α when administered in luteal cells activates RAF/ERK/MAPK kinase pathway which subsequently up regulates EGR1 which in turn induces *TGFβ1* expression [17]. In luteal cells, PGF2α also in different signaling pathway stimulates thrombospondins 1 which activates TGFβ1 production [44]. In support of this, expression of TGFβ1 has been found to be reduced when TSP1 was silenced in bovine corpus luteum [45]. On the other hand, binding of PGF2α to its receptor increases free intracellular calcium and also enhances the activity of mitogen-activated protein (MAP) kinase, phospholipase C and protein kinase C (PKC), with subsequent activation of multiple transcription factors [5, 46] including the ATF3, which plays an important role in the regulation of CL regression through production of TGFβ1, through a process independent of EGR1 in cattle [47]. In another study conducted in rats, the PGF activated JNK/SAPK signaling pathway stimulates the production of AP1 transcription factor that contribute to luteal regression via induction of chemokines including TGFβ1, also without the involvement of EGR1 [48]. These could be the plausible explanations why PGF2α induced TGFβ1 production in our study was found to be dysregulated with EGR1 that is sharp contrast with the findings in cattle [17].

The present study also showed that the mRNA expression of *Caspase 3* was significantly up regulated in EGR1 KO luteal cells, and PGF2α treated KO luteal cells. Early growth response genes are important transcriptional regulators linked to cell proliferation and survival [26, 49, 50]. The protein EGR1 directly or indirectly influences gene expression that is essential to cell proliferation [51]. Based on the cell environment and the primary stimuli employed, EGR1 has both pro-survival and pro-apoptotic activities [13]. The reduction in progesterone concentration in spent media in EGR1 KO luteal cells and PGF2α treated EGR1 KO luteal cells in our study might be due to the increase apoptosis, reduction in the number of steroidogenic cells and a degeneration of the capillary network of the mature corpus luteum. Furthermore, significant up regulation of Caspase 3 and subdued progesterone production during in vitro treatment of EGR1 KO luteal cells with PGF2α might be due to pronounced inhibition of EGR1 mediated luteal cell proliferation and migration. TGFβ1 production is known best for its potential involvement in apoptosis and tissue remodeling [42] and decreases progesterone secretion in luteal cell cultures [19]. Thus, the present study provides an important insight on functional role of EGR1 in Prostaglandin F2 alpha induced TGFβ1 expression during luteal regression in buffaloes.

## Conclusion

The present study provided new insight into how the buffalo CL responds to PGF2α. It also illustrated some of the signaling pathways involved in regulating various functional and structural changes that occur during luteal regression. In our study, we have demonstrated that the luteolytic dose of PGF2α induced TGFβ1 expression via EGR1 independent mechanism in buffalo. Despite the acquired expertise, much remains to be learned. Future studies elucidating the underlying mechanism of PGF2α induced TGFβ1 expression during luteal regression will contribute to improve assisted reproductive technologies.

## Data Availability

All data generated or analysed during this study are included in this published article.

## Abbreviations

CL: Corpus Luteum
EGR: Early growth response
PGF2α: Prostaglandin F2 alpha
TGF β1: Transforming growth factor β1
